# The Flowering of Positive Psychology in Foreign Language Teaching and Acquisition Research

**DOI:** 10.3389/fpsyg.2019.02128

**Published:** 2019-09-24

**Authors:** Jean-Marc Dewaele, Xinjie Chen, Amado M. Padilla, J. Lake

**Affiliations:** ^1^Department of Applied Linguistics and Communication, University of London, London, United Kingdom; ^2^Graduate School of Education, Stanford University, Stanford, CA, United States; ^3^Department of English, Fukuoka Jo Gakuin University, Fukuoka, Japan

**Keywords:** emotion, positive psychology, foreign language acquisition, language learners, language teachers

## Abstract

The present contribution offers an overview of a new area of research in the field of foreign language acquisition, which was triggered by the introduction of Positive Psychology (PP) (MacIntyre and Gregersen, 2012). For many years, a cognitive perspective had dominated research in applied linguistics. Around the turn of the millennium researchers became increasingly interested in the role of emotions in foreign language learning and teaching, beyond established concepts like foreign language anxiety and constructs like motivation and attitudes toward the foreign language. As a result, a more nuanced understanding of the role of positive and negative learner and teacher emotions emerged, underpinned by solid empirical research using a wide range of epistemological and methodological approaches. PP interventions have been carried out in schools and universities to strengthen learners and teachers’ experiences of flow, hope, courage, well-being, optimism, creativity, happiness, grit, resilience, strengths, and laughter with the aim of enhancing learners’ linguistic progress. This paper distinguishes the early period in the field that started with MacIntyre and Gregersen (2012), like a snowdrop after winter, and that was followed by a number of early studies in relatively peripheral journals. We argue that 2016 is the starting point of the current period, characterized by gradual recognition in applied linguistics, growing popularity of PP, and an exponential increase in publications in more mainstream journals. This second period could be compared to a luxuriant English garden in full bloom.

## Introduction

Emotions are the heart of language learning and teaching, and yet they have largely remained in the shadows in the past decades of applied linguistic research. Swain (2013) argued, “emotions are the elephants in the room – poorly studied, poorly understood, seen as inferior to rational thought” (p. 195). Applied linguists may have underestimated the relevance of emotions in the past decades because of the dominance of cognitive perspectives (Sharwood Smith, 2017) and the false belief that the study of emotion is somehow unscientific. The situation is changing rapidly. Mackenzie and Alba Juez (2019) note that “across social sciences, scholars are recognizing the essential role of emotional phenomena” (p. 3) and they label this emerging interdisciplinary field as “emotionology” (back cover). This view concurs with the one in Prior’s (2019) position paper on emotion in *The Modern Language Journal.* He argues that scholarly interest in the emotional dimensions of language learning, teaching, and use is booming and that it is about time to acknowledge the presence of the elephant in the room. The time has come to study it in new ways in order to “open up this confined and crowded room and explore other spaces of language and emotional life” (p. 525). In their commentary on Prior’s (2019) position paper, Lantolf and Swain (2019) observe that interest in emotion theory and research is spreading across disciplinary boundaries. Research on learner and teacher emotions is also reaching into the political sphere by focusing on hegemonic power relations (Benesch, 2017, 2018). In his own commentary of Prior’s (2019) position paper, Dewaele (2019a) combined the elephant in the room metaphor with the adynaton “when pigs fly” (i.e., something that will never happen) to describe the current interest in emotion as the time of the flying elephants. It is important to point out that affect and emotion have figured more centrally in more teacher-oriented research since the turn of the century (Arnold, 1999; Avila-López, 2015).

Describing developments in a field in full expansion, while respecting a strict word limit, is a daunting exercise. We will start with a brief description of Positive Psychology (PP)^1^ before adopting a broad chronological approach to describe the emergence of PP in applied linguistics. We distinguish two periods in which PP moved from the periphery to a more central position in the field. We decided to adopt a moderate level of granularity, including not just the most influential contributions but also those whose influence may only be felt in the future. Rather than appraising contributions critically on an individual basis, we opted for broad critical considerations at the end of the period, concluding with suggestions for further research. We will set the general academic context for the two periods, before distinguishing theoretical contributions (research agendas, theoretical considerations, overviews, assessment practices), empirical studies on learners and teachers, and PP intervention studies.

## Positive Psychology

The growing popularity of Positive Psychology (PP) in the last two decades has caused a powerful shift away from an exclusive focus on problems in general psychology. PP originated with a call in the second part of the twentieth century to pay more serious attention to the positive side of life (Lopez and Snyder, 2009, p. 4), though “In one sense, positive psychology is thousands of years old, dating back to the thoughts of ancient philosophers and religious leaders who discussed character, virtues, happiness and the good society” (Diener, 2009, p. 7). PP is the empirical study of how “normal” people live with the goal of helping them to thrive and flourish (Seligman and Csikszentmihalyi, 2000; Peterson, 2006; Csikszentmihalyi and Nakamura, 2011).

PP researchers do not deny the existence of problems, but complement them with “positive” topics such as flow, hope, courage, well-being, optimism, creativity, happiness, flourishing, grit, resilience, positive emotions, life longings, emotional creativity, strengths, wisdom, health, laughter (Lopez and Snyder, 2009). Seligman and Csikszentmihalyi (2000, p. 5) pointed out that PP research is founded on three main pillars: “positive subjective experience, positive individual traits, and positive institutions.”

PP has had a unifying effect in the field as old agendas were put aside and psychologists from different backgrounds with common interests came together to focus on things that matter to “normal” people (Lopez and Gallagher, 2009, p. 4). Interdisciplinary perspectives are key to this research as well as science-practice integration: “practitioners are always either implementing empirically supported protocols, or helping generate the empirical basis for new programs. In this way, we could ensure that PP interventions remain firmly in the realm of science rather than pseudoscience” (p. 6).

Seligman et al. (2009) carried out an 18-month-long PP intervention after randomly assigning 347 year 9 students to Language Arts classes that contained a PP curriculum and a control group that did not have the PP curriculum.

PP exercises included instructing students “to write down three good things that happened each day for a week” and “helping students identify character strengths in themselves and others, using strengths to overcome challenges, and applying strengths in new ways” (p. 301). Compared to the control group, the group who had the PP intervention reported more enjoyment and engagement in school “(curiosity, love of learning, creativity),” and mothers and teachers reported, “improved social skills (e.g., empathy, cooperation, assertiveness, self-control)” (p. 301). The researchers then instructed 100 staff members of a school in Australia on the principles and skills of PP with the aim of raising the well-being of all students. They report, “the programme was enormously successful” (p. 304).

Seligman (2018) hypothesized that [positive emotion (P), engagement with activities that use one’s character strengths (E), developing positive interpersonal relationships (R), finding meaning by serving a cause beyond oneself (M), and recognizing areas of accomplishment and achievement (A)] PERMA are the building blocks of well-being, which is the basis for flourishing. Individuals in such a state thrive, feel full of vitality, and prosper both at an individual and a group level. Seligman and Csikszentmihalyi (2000) positioned PP as a rigorous scientific approach: “Positive psychology does not rely on wishful thinking, faith, self-deception, fads, or hand waving; it tries to adapt what is best in the scientific method to the unique problems that human behavior presents to those who wish to understand it in all its complexity” (Seligman and Csikszentmihalyi, 2000, p. 7).

## Positive Psychology in Applied Linguistics

### New Developments in the Periphery (2012–2015)

#### Context

One could argue that the seeds of PP fell on fertile ground in applied linguistics. Research into “affective” factors had blossomed, and many researchers focused on attitudes, motivation, and foreign language anxiety, which clearly had an emotional dimension, albeit slightly hidden from view, with a strong bias toward negative emotions (anxiety). Educational psychologists and teacher trainers had emphasized the importance of positive affect in foreign language (FL) classes (Arnold, 1999; Arnold and Fonseca, 2007; Arnold and Fonseca Mora, 2011) and the role of emotions in FL learning had been highlighted (Dewaele, 2005; MacIntyre et al., 2009; Bown and White, 2010; Puozzo Capron and Piccardo, 2013; Dewaele, 2015), but overall this type of research had remained somewhat in the shadow in a field dominated by a cognitive perspective (Sharwood Smith, 2017) and no connection had been established with PP.

#### Theoretical Contributions

Although a number of early papers had adopted PP variables in applied linguistic research (Egbert, 2003, 2004;^2^ Tardy and Snyder, 2004; Rubio, 2011), the first paper to explicitly introduce PP research in applied linguistics was, not surprisingly, co-authored by a prominent psychologist and an applied linguist with a strong interest in foreign language (FL) teaching (MacIntyre and Gregersen, 2012). They refer to Fredrickson’s (2001, 2003, 2006) “broaden and build” theory of emotions and its associated action tendencies. They report the five functions of positive emotions as follows:

First, positive emotions tend to broaden people’s attention and thinking, leading to exploration and play, new experiences and new learning. Second, positive emotion helps to undo the lingering effects of negative emotional arousal. A related, third function of positive emotion is to promote resilience by triggering productive reactions to stressful events, such as improving cardiovascular recovery and making salient feelings of happiness and interest while under stress. Fourth, positive emotion promotes building personal resources, such as social bonds built by smiles, intellectual resources honed during creative play, and even when young animals practice self-preservation maneuvers during rough-and-tumble play. Fifth, positive emotions can be part of an upward spiral toward greater wellbeing in the future, essentially the vicious cycle in reverse (MacIntyre and Gregersen, 2012, pp. 197–198).

Because emotions are semi-controllable, MacIntyre and Gregersen (2012) argued that teachers have the potential to influence students’ emotions by appealing to their imagination and to help them notice the gap between their current and future selves. Teachers also need to create a safe environment where the influence of negative emotions is reduced. They proposed the technique to encourage students to use their imagination to modify negative emotional schema: “to reduce the intensity of conditioned negative-narrowing emotional responses that individuals associate with language learning by replacing it with a relaxation response when confronted with the negatively conditioned stimulus” (p. 205). The aim is not so much the complete absence of negative emotions, but rather the harnessing of the power of positive emotions in order to create a balance. Indeed, joy, interest, contentment, pride, and love allow students to learn better as they enhance their ability to notice things in the classroom environment and strengthen their awareness of language input. Being in a positive emotional state allows students to absorb the FL better and to erase the after effects of negative emotions. Finally, positive emotions help build students’ longer term resiliency and hardiness to overcome future negative events. Gregersen (2013) explained that PP could be hugely beneficial for FL teachers and learners who could capitalize on positive affect while mitigating the effect of negative emotions, i.e., anxiety.

A special issue on PP, guest edited by MacIntyre and Gregersen, grew out of a symposium at the 2014 *International Conference for Language and Social Psychology* in Hawaii. MacIntyre and Mercer (2014, p. 154) refer to the saying that as a scholarly area “PP has been said to have a short history and a long past.” Identifying the early adopters of PP in applied linguistics, MacIntyre and Mercer (2014) referred to Lake (2013) who “was one of the first to explicitly adapt and apply PP concepts” (p. 158) in his study of Japanese learners’ positive self, positive English L2 self, self-efficacy, and intended effort. One could add that traces of positive emotion were already present in Horwitz et al.’s (1986) Foreign Language Classroom Anxiety scale with the item “I feel confident when I speak in English class,” which was to be reversed to reflect anxiety.

MacIntyre and Mercer (2014) also considered the criticism aimed at PP, especially that formulated by Lazarus (2003) who disliked “(1) the over-use of cross-sectional research designs, (2) a tendency to treat emotion too simplistically as either positive or negative, (3) inadequate attention to both differences among individuals within a group as well as the overlap between groups when discussing statistically significant group differences, and (4) poor quality measurement of emotions” (p. 160). MacIntyre and Mercer (2014) argued that Second Language Acquisition (SLA) researchers might have an advantage over psychologists:

In future studies, SLA research might be at an advantage over the discipline of psychology, having travelled much further down the road of recognising the genuine value in research that allows a variety and mixture of epistemological and methodological stances. SLA research has developed an openness to different understandings of empirical studies such as those employing systematic, rigorous qualitative research (p. 161).

Addressing Lazarus’ (2003) criticism of cross-sectional designs and simplistic distinction between positive and negative emotions, MacIntyre and Mercer (2014) encourage researchers to look at emotions over different time scales, from the short term in seconds to the long term in years, and they point to the concept of “ambivalence” in SLA, where learners can simultaneously experience a positive and a negative emotion. They also point to the social turn in SLA that has highlighted the crucial importance of contexts in SLA. Next, they point to the emerging interest in complex dynamic systems that is particularly useful in PP-inspired applied linguistic research, where multiple variables interact and fluctuate dynamically. Finally, they argue in favor of a combination of group-level perspectives as well as dense individual perspectives “that can describe in some detail the processes that lead to happiness, the protective force of learned optimism, or describe the most enjoyable facets of learning for a specific person” (MacIntyre and Mercer, 2014, p. 166).

The organization of the first conference on the *Psychology of Language Learning* (PLL) by Sarah Mercer at the University of Graz in 2014 also consolidated a fledging field. It gave veteran and younger researchers who had been working in this area the opportunity to meet and discuss PP concepts and to generate interest in the PP approach.

#### Empirical Studies

Among the contributions in MacIntyre and Mercer’s (2014) special issue, Oxford and Cuéllar (2014) used Seligman’s (2018) PERMA model to analyze the narratives of five university students learning Chinese in Mexico. A grounded-theory approach allowed them to identify the themes that did not entirely overlap with PERMA, namely “(1) emotions, (2) unification of engagement and meaning, (3) relationships within and across cultures, and (4) accomplishment” (p. 183).

Answering similar questions, but adopting a quantitative approach, Chaffee et al. (2014) investigated how 100 Canadian university FL students managed to sustain their motivation and love of the FL in a negative learning environment. Resilience and positive reappraisals were found to be the key to enjoy a difficult language learning experience with a controlling teacher.

In a large-scale mixed-methods study, Dewaele and MacIntyre (2014) used an online questionnaire to collect quantitative and narrative data from 1,746 FL learners from all over the world about their FL Enjoyment (FLE) and FL Classroom Anxiety (FLCA). They established that these are essentially separate emotion dimensions. Levels of FLE were higher – and FLCA lower – among more advanced students and those who felt that they performed above the group average. Cultural background and age also had an effect on FLE and FLCA with North American participants and older learners reporting more FLE and less FLCA while Asian participants and younger learners reported lower FLE and higher FLCA. Qualitative analysis of the feedback on the most enjoyable episodes in the FL class revealed that specific classroom activities that involved some degree of autonomy were the most frequently mentioned sources of FLE. Teachers who were supportive, positive, well organized, and happy and who could be funny and respectful of students boosted students’ FLE.

In a study that included both FL learners and teachers, Gregersen et al. (2014) adopted a qualitative approach to examine how emotional intelligence played a role on perceived growth in the attainment of L2 possible selves among 19 American university students and pre-service TESOL teachers in a series of PP interventions over a 3-week period. A focused analysis of two participants, one student and one pre-service TESOL teacher, showed that they used emotional intelligence to deal with stressful situations and managed “to understand and integrate their experiences inside and outside the classroom as part of the language learning and teaching process” (p. 328).

Singing was at the heart of the PP intervention described in Murphey (2014). He reported how he taught his 155 Japanese students short English affirmation songlet-routines they would sing to others out of the classroom over a 4-year period. He found that “when we share important information we all can become more ‘well’ especially when that sharing involves us singing it together” (p. 225).

Falout (2014) argued that spatial factors in the classroom could contribute to a positive atmosphere. More specifically, circular seating arrangements that increase proximity to the teacher can boost a sense of trust, empathy, and belonging in the group of language learners as it creates an all-inclusive social action zone for the whole class.

Aguilar-Río (2013) combined classroom observation and subsequent interviews with one language teacher in order to understand the thoughtful planning and the emotional responses underlying her actions in an institutional context. Similarly, Gabryś-Barker (2014) presented a qualitative study of 50 EFL trainee teachers narratives and their perceptions of teacher enthusiasm and the positive impact it has through emotional contagion on teaching and learning success. She presents a list of verbal and non-verbal strategies that trainee teachers could adopt to overcome setbacks and become enthusiastic teachers.

#### Critical Considerations

We are aware that our review is not exhaustive, but it represents a sample to illustrate the rapid emergence and burgeoning of the new research area. In this early period, the majority of these PP studies linked positive (or negative) emotions to SLA or teaching with the aim of identifying their specific effects. It is striking that most designs were cross-sectional reflecting the first concern of Lazarus (2003) about previous work in PP. Dörnyei (2007) made a similar point about the preponderance of cross-sectional research in applied linguistics. Longitudinal research allows researchers to describe change over time and to dig into causality (p. 78). However, Dörnyei acknowledges that the amount of time and effort longitudinal research requires makes it less attractive for researchers who are under constant pressure to publish (p. 88). Moreover, it is easier to recruit participants for cross-sectional studies where only a single effort is required, in contrast with longitudinal designs where multiple data collection leads to considerable participant attrition. One consequence of the snapshot approach in the quantitative studies is that it is difficult to pinpoint causation. Considering Lazarus’ (2003) second point about the simplistic categorization of emotions as either positive or negative, we would argue that valence being a universal dimension (Frijda, 1986), it is a legitimate way to categorize emotions, while remembering that ambivalence exists and that emotions can fluctuate quickly (MacIntyre and Mercer, 2014). Lazarus’ (2003) third point about lack of attention to intra-group differences does equally not apply to the studies we reviewed so far, where no single group was considered as homogeneous, and where the voices of participants were heard in the mixed methods and/or qualitative studies. Lazarus’ (2003) final point about poor-quality measurement of emotions does not apply to the qualitative studies, but the psychometric qualities of quantitative research instruments were to be investigated in more detail in the next period. One distinguishing feature of the work in the early period is that it happened very much in the periphery. In other words, major journals were more likely to reject the new work, even if they had accepted work by the same authors on more traditional topics. Similarly, some of the work developed in the period was presented at major conferences, such as the annual conference of the *American Association of Applied Linguistics*, but proposals for panels on PP at the same conference were turned down.

### The Flowering of PP Research in Applied Linguistics (2016–Present)

#### Context

Symbolically, one could argue that PP penetrated mainstream applied linguistics research with the organization of the second PLL conference at the University of Jyväskylä in 2016, where plans were made for the establishment of the *International Association for the Psychology of Language Learning* – which happened at the PLL3 conference at Waseda University in Tokyo in 2018. Also, the publisher, Multilingual Matters, agreed to establish a new book series entitled *Psychology of Language Learning and Teaching* with Sarah Mercer and Stephen Ryan as series editors, to give a unique place to contributions that would have otherwise have been placed in the more generic SLA book series. Two edited books in 2016 also heralded the official arrival of PP in mainstream applied linguistics. The first book was entitled *Positive psychology in SLA* (MacIntyre et al., 2016) with contributions that focused on “what makes language learning meaningful and fulfilling” (p. 4) The book editors conclude that “Positive psychology has an added dimension of practice and applications that can further inform both the teaching and learner development sides of SLA” (MacIntyre et al., 2016, p. 378). The second book, edited by Gabryś-Barker and Gałajda (2016) was entitled *Positive psychology perspectives on foreign language learning and teaching* and contained contributions by MacIntyre, Oxford, Gregersen, and Mercer as well as a number of prominent Polish researchers. From 2017, papers adopting a PP perspective in applied linguistics started appearing in more established and prestigious applied linguistic journals. Until then, new online journals such as *Studies in Second Language Teaching and Learning* had provided a perfect platform for the fledging area. As applied linguists grasped the potential that a PP perspective offered to their research, its popularity increased, its status grew, and what had until then been the early flowers announcing the arrival of spring, turned into an outburst of color and sweet smells of ever-expanding flower beds. A number of special issues and edited books appeared on learner emotions, and on learner and teacher psychology (Agudo, 2018; De Costa et al., 2018; Li, in press; Mercer and Kostoulas, 2018; Dewaele and Li, 2018; Dewaele, 2018; Berdal-Masuy and Pairon, 2019), to the point that White (2018) talked about the emotional turn in applied linguistics and TESOL, complementing Pavlenko’s (2013) affective turn.

From 2016, the epistemological and methodological range of PP research in applied linguistics and TESOL expanded rapidly and further connections were established with existing concepts and theories on motivation. New dependent and independent variables were included in research designs.

#### Theoretical Contributions

Oxford (2016a) offered a general introduction of PP to applied linguists, using PERMA as a starting point for the development of her own EMPHATICS vision. The acronym stands for “Emotion and empathy, Meaning and motivation, Perseverance, including resilience, hope and optimism, Agency and autonomy, Time, Hardiness and habits of mind, Intelligences, Character strengths, Self factors (self-efficacy, self-concept, self-esteem, self-verification)” (Oxford, 2016a, p. 11).

Mercer (2016) focused on empathy in FL teaching and learning, arguing that it plays a central role in interpersonal skills and social relationships in the classroom. Indeed, empathy fosters appreciation of foreign cultures and peers among learners, and it allows teachers to engineer positive group dynamics that create an optimal classroom atmosphere. Falout (2016) followed a similar route, drawing on PP to investigate learners’ past and imagined future selves in order to allow learners to reflect on their FL learning experience, to nurture positive emotion, and to build greater engagement, adaptability, and self-consistency.

Kusiak-Pisowacka (2016, p. 289) reflected on ways to implement PP principles in FL testing, with the aim of making “evaluation a positive experience for both learners and teachers.” By adopting a positive approach to testing, and using less traditional techniques such as think-aloud protocols, interviews and conferencing with students, evaluation can become “a fruitful constructive learning/teaching situation” (p. 289).

MacIntyre et al. (2019a) set out an ambitious agenda for PP in applied linguistic research and sought to allay fears that PP focused exclusively on the positive. Their first point is that PP can strengthen the field by encouraging researchers to acknowledge that there are interactions between positive and negative phenomena and that equating the positive to “good/motivated/successful” and negative to “bad/unmotivated/unsuccessful” is simplistic as “language learners’ and teachers’ emotional and psychological experiences are complex and often conflicted” (p. 269). A second point is a resolute rejection of the deficit model in learners and teachers. Rather than obsessing about what FL learners and teachers lack, MacIntyre et al. (2019a) argue that it would be more fruitful to look at strengths and opportunities – without denying that problems may exist. Their third point is an acknowledgement that FL learning involves more than just a balancing of the positive and the negative at an individual level: “The complexity also extends to a perception of the learner as an individual who is set into a sociocultural context. Language learning and teaching are by their very nature intercultural experiences” (p. 269). In terms of epistemology, they argue in favor of “empirical and theoretical plurality” (p. 269), encouraging quantitative, qualitative, and mixed-method research, preferably with scholars and practitioners joining forces. They call for “programmatic research on both the psycho-social and language development effects of PP Interventions” (p. 269).

#### Empirical Learner-Focused Studies

Since 2016, studies that focused on the first two pillars of PP (c.f., Seligman and Csikszentmihalyi, 2000) namely positive emotions and positive character traits have bloomed. Variables such as empathy, selves, flow, perseverance, motivation, engagement, perseverance, love, and passion have attracted widespread attention. One vibrant area that has expanded geographically and methodologically since its inception in the first period is the research on learners’ FLE and FLCA. What these studies have in common is a desire for the findings to lead to improved educational practices allowing teachers to optimize the emotional climate in their FL classrooms in order to foster linguistic progress and well-being.

Self-concept was at the heart of Lake’s (2016) investigation into a group of 212 first-year Japanese female students in a private university in Japan. Using Structural Equation Modeling, he found that characteristics at a global level, referencing the whole person, such as a positive self-concept, are not necessarily directly linked to L2 proficiency, but rather to an intermediate domain level where positive L2 self and L2 self-efficacy are located.

Ibrahim (2016) adopted a phenomenological approach to focus on enjoyment, directed motivational currents, and long-term engagement of 7 FL learners. The main source of happiness in learning the L2 came from the transformational process of personal growth, including skills, image, and identity.

Following up on previous studies on flow, Czimmermann and Piniel (2016) looked into Hungarian FL learners’ experiences of flow and anti-flow (anxiety, boredom, and apathy) and found that the key for obtaining flow experiences is providing learners with sufficient time to build concentrated engagement with motivating tasks that are difficult, but manageable, and giving learners sufficient autonomy to execute them without teacher interference. Flow and anti-flow were also the focus of Dewaele and MacIntyre’s study (in press) on 232 Spanish FL learners from around the world. Participants were found to experience significantly more flow than anti-flow in their classroom. Percentage of time in a state of positive flow was positively linked to a higher degree of multilingualism, high relative standing in the group, age, and number of years of FL study.

Belnap et al. (2016) focus on boosting the perseverance of 52 American language students by increasing their self-efficacy and self-regulating abilities during an intensive Arabic program in Jordan where they faced communication challenges. Material was collected through journals, interviews, and oral proficiency tests. Progress in proficiency was found to be positively linked to satisfaction with speaking Arabic.

Enjoyment and love were the focus of Pavelescu and Petrić’s qualitative investigation (2018) into the experiences of four high school EFL learners in Romania. Two participants experienced strong and stable love toward English, while the other two participants reported enjoyment in their English language learning without being in love with English. Pavelescu and Petrić (2018) argued that love served as the fuel for the learning process, as it allowed learners to create effective coping mechanisms when some classes were not enjoyable and it helped them invest greater effort into the learning and the use of English in and out of the classroom. Passion was also the focus of the quantitative empirical study by Chen et al. (2019) with 260 high school L2 learners in Taiwan. The authors developed a process model linking passion and adaptive outcomes both *in* L2 as well as *outside* in one’s life in general. Based on the Dualistic Model of Passion findings, demonstrated that being passionate about L2 learning provided positive benefits for both the learning of an L2 as well as heightening the well-being of learner in general. In a recent study on bilingual U.S. college students, Chen and Padilla (2019) found four important components (emotional, social, psychological, and linguistic) that are central to well-being and which contribute to flourishing.

Arguing that researchers should also consider the emotional experiences of language students outside the classroom, Ross and Rivers (2018) collected data through semi-structured interviews from eight university ESL learners in Australia. Ross and Rivers (2018) found that their participants’ emotional experiences in English beyond the classroom were more intense than those inside the classroom.

Researchers have also been looking for sources of FLCA and ways to alleviate FLCA (Oxford, 2017). Jin and Dewaele (2018) considered the effect of learners’ positive orientation and perceived teacher and student emotional support on FLCA of 144 Chinese EFL university students. Statistical analyses revealed that positive orientation was linked to significantly lower FLCA. However, while stronger perceived teacher support did not significantly lower levels of FLCA, stronger emotional support from peers was linked to lower levels of FLCA.

Dewaele and MacIntyre (2016) re-analyzed their 2014 corpus using Principal Components Analysis. They use the metaphor of left and right feet to describe learners’ FLE and FLCA. This mixed-method study allowed them to identify two sub-dimensions of FLE, namely social and private FLE. Analysis of qualitative data showed that risk was inherent in enjoyable episodes and that FLE and FLCA do not behave in a seesaw manner, where the absence of one automatically boosts the presence of the other. Early studies on FLE and FLCA focused exclusively on the effect of learner-internal variables (Dewaele and MacIntyre, 2014). More recent work has also included learner-external variables in the research design in order to see which are better predictors of FLE and FLCA. Moreover, data were collected from single contexts (city or country) in order to have more homogeneity in the linguistic and cultural profiles of the participants and in the target languages. These latest studies pay particular attention to the dynamic interactions among a wide range of independent and dependent variables.

One such context-specific study was that of Dewaele et al. (2018b) who collected data on FLE and FLCA from 189 secondary school pupils in two schools in Greater London who had English as an L1 and were mostly studying French as a FL. A weak negative correlation was found between FLE and FLCA. Attitudes toward the teacher and teacher practices were found to have a much stronger effect on FLE than on FLCA. FLCA was linked to negative attitudes toward the FL, lower relative standing in the peer group, and being less advanced in the FL. In contrast, FLE was strongly predicted by positive attitudes toward the FL, positive attitudes toward the teacher, frequent use of the FL by the teacher, a larger proportion of time spent on speaking during classes, a higher relative standing in the peer group, and being more advanced in the FL.

In a follow-up study, Dewaele and Dewaele (2017) used a pseudo-longitudinal design to see whether levels of FLE and FLCA, and their predictors, remained stable during secondary education. A comparison of the 12–13 year olds, the 14–15 year olds, and the 16–18 year olds revealed that FLCA remained unchanged while FLE increased slightly over time. A different set of independent variables predicted FLE and FLCA in the three age groups. In the youngest group, FLE was predicted by peers and FLCA by the self. In the middle group, FLE depended more on the teacher while FLCA was again predicted by the self. In the oldest group, FLE was very strongly predicted by the teacher while FLCA was predicted by peers. It thus seems that limited changes in mean levels of FLE and the stability in FLCA hid dynamic interactions between various psychological and sociobiographical variables in shaping learners’ FLE and FLCA.

In a final follow-up study, Dewaele and Dewaele (in press) investigated to what extent FLE and FLCA vary at a single point in time when facing two different teachers for the same FL. Participants were a subgroup extracted from the complete sample, namely 40 students who had one main teacher and a second teacher for the same FL. FLCA was found to be constant with both teachers, but students reported significantly higher FLE with the main teacher. This corresponded with significantly more positive attitudes toward the main teacher, more unpredictability, and more frequent use of the FL in class by the main teacher, which are all predictors of FLE. Classroom-specific items linked to the teacher interventions to create a positive emotional atmosphere contributed to higher FLE scores. Items reflecting more stable personal and group characteristics varied much less between the two teachers. Dewaele and Dewaele (in press) conclude that FLE is a more fleeting classroom emotion while FLCA is more stable.

In addition to studies developed in Western contexts, an increasing number of studies on FLE and FLCA have been conducted in Asian countries. Intrigued by lower FLE and higher FLCA among Asian FL learners reported in Dewaele and MacIntyre (2014), researchers focused specifically on the uniqueness of FLE and FLCA in the Chinese context. Li et al. (2018) developed a Chinese Version of the FLE Scale and collected data from 2,078 Chinese high school students. Three factors emerged from a Principal Component Analysis: FLE-Private, FLE-Teacher, and FLE-Atmosphere. Participants reported that the teacher and to a lesser degree peers shaped their FLE.

Following this avenue of research, Li et al. (2019) investigated the relationship between FLE, FLCA, and EFL achievement of 1,307 Chinese EFL university students. A significant negative link emerged between FLCA and self-perceived EFL proficiency while FLE was significantly, positively, linked to self-perceived EFL proficiency, confirming earlier research (Piechurska-Kuciel, 2017; Dewaele and Alfawzan, 2018). The strength of the relationship between emotions and self-perceived EFL proficiency in Li et al. (2019) depended on the participants’ proficiency level. FLE was a stronger positive predictor of self-perceived EFL proficiency than FLCA in the low proficiency group, where participants reported more FLCA and less FLE. In the medium and high proficiency groups, FLCA became a stronger predictor of self-perceived EFL proficiency. Participants reported that disappointing English test results and harsh criticism by the teacher inflated their FLCA while good test results, friendly words from the teacher, and good social standing boosted their FLE.

Similar patterns between FLE and FL achievement emerged in Jin and Jun Zhang’s (2018) study of 320 Chinese EFL high school students. A three-factor solution emerged from a factor analysis of an adapted FLE scale: Enjoyment of teacher support, Enjoyment of student support, and Enjoyment of FL learning. FLE exerted both direct and indirect effects on students’ achievement scores. Enjoyment of FL learning had the strongest effect on FL achievement with enjoyment of teacher support and enjoyment of student support having an indirect effect. The same authors developed a shorter version of their previous Chinese version of the Foreign Language Enjoyment Scale (Jin and Jun Zhang, 2019) claiming it showed a more solid dimensional division and better psychometric properties than Li et al.’s (2019) scale.

In another study with Chinese EFL learners, Jiang and Dewaele (2019) investigated to what extent levels and sources of FLE and FLCA of 564 Chinese students differed from FL learners outside China. While mean levels of FLE were found to be quite similar, FLCA levels were higher than in Dewaele and MacIntyre (2014). Relationships between learner-internal, teacher-related variables and levels of FLE and FLCA were generally comparable to those identified outside China, with the exception of a positive relationship between Chinese students’ FLE and teachers’ predictable behavior.

Stressing the importance of looking at other target languages beyond English, Dewaele et al. (2019b) focused on 592 learners of Turkish as a FL in Kazakhstan. Kazakh students’ levels of FLE and FLCA in Turkish classes were found to be broadly similar to those reported in previous research. FLE in Turkish was found to be strongly predicted by attitude toward Turkish, followed by teacher-centered variables with little effect of learner-internal variables. The only slight difference with previous studies was that FLCA was weakly predicted by some learner-internal as well as teacher-centered variables.

The effect of the teacher on FLE and FLCA of 210 Spanish EFL students was the exclusive focus of Dewaele et al. (2019a). Teacher characteristics were found to explain more than twice as much variance in FLE than in FLCA. The strongest predictor of FLE was teacher’s friendliness while the teacher’s strong foreign accent in English lowered students’ FLE. Participants reported more FLCA with younger teachers, teachers who were overly strict, and teachers who used little English in class.

The influence of political and historical context and the effect of the target language were at the heart of the investigation of De Smet et al. (2018) on the FLE and FLCA of 896 Belgian francophone primary and secondary school pupils. They compared two target languages (English and Dutch) in two different types of school in francophone parts of Belgium. The first type was regular school where students were taught in French and where they had FL classes of Dutch and English. The second type was a school that had adopted Content and Language Integrated Learning (CLIL) and where some content classes were taught in Dutch or English. De Smet et al. (2018) found that CLIL pupils experienced significantly less FLCA than their non-CLIL peers, but that levels of FLE were similar. English elicited significantly less FLCA and more FLE than Dutch, which suggests that the historical and political context, and more specifically inter-group relations between the francophone and Dutch-speaking communities, as well as the type of school system, shaped FL learners’ emotions.

In a study based on cross-sectional and longitudinal data from 108 Japanese EFL pupils, Saito et al. (2018) investigated to what extent FLE, FCLA, and motivation affected the development of comprehensibility in English over a period of 3 months. A factor analysis unveiled a three-factor solution similar to that reported in Dewaele and MacIntyre (2016) with FLCA, Social FLE, and Private FLE. Levels of Private FLE (but not FLCA) and a clearer vision of ideal future selves were significantly positively correlated with English use both inside and outside of the English classroom and with pupils’ total frequency of English conversations. Students with the largest gains in comprehensibility in English reported significantly more Private FLE and less FLCA. An important finding was that Private FLE and Ideal L2 Self were independent predictors of gains in comprehensibility.

The following studies focused specifically on dynamic fluctuations and change in FLE and FLCA. Firstly, Boudreau et al. (2018) adopted the idiodynamic approach to measure fluctuation in FLE and FLCA on a second-by-second basis for about a minute. Ten Anglo-Canadian students completed speaking tasks in French L2 after which they viewed the recording of their performance and reported their levels of FLE and FLCA. Subsequent interviews about the reasons for the fluctuations allowed the researchers to understand local causes. Correlation analyses of the multiple FLE and FLCA values of each participant revealed that these veered from positive to negative and then to zero. High FLE momentarily coincided with low FLCA, but this relationship could shift completely a few seconds later. It confirmed the view that FLE and FLCA are independent dimensions. Participants explained in the interview that the fluctuations could be linked to difficulties in word searches, to momentary failure to control FLCA, to enjoyment or boredom in discussing a particular aspect of the task.

Dewaele and Dewaele (2018) adopted a more traditional quantitative perspective to measure the effect of FLE and FLCA on Willingness to communicate (WTC) of 189 British secondary school pupils who were studying a FL in London. FLCA turned out to be the strongest negative predictor of WTC. Weaker positive predictors of WTC were frequent FL use by the teacher, a positive attitude toward the FL, social FLE, and age. They concluded that teachers play a key role in boosting learners’ WTC by generating a positive and supportive emotional classroom. Moreover, fostering linguistic and cultural interest in the FL encouraged learners to seize the opportunity to use the FL in front of their peers and teacher.

The same research questions guided Dewaele’s (2019b) study on the predictors of WTC of 210 Spanish EFL learners. Here also, FLCA turned out to be the strongest (negative) predictor of WTC, explaining twice as much variance as FLE and teacher’s frequency of use of the FL that were positive predictors of WTC.

Further research into the unique nature of FLE and FLCA explored the role of personality traits. Dewaele and MacIntyre (2019) collected data about FLE and FLCA from 750 FL learners from around the world. They found that FLCA was strongly predicted by the personality trait Emotional Stability and less so by Social Initiative. In contrast, FLE was strongly predicted by teacher-centered variables and less so by the personality trait, Cultural Empathy. Considering the relative effect of personality traits on FLCA and FLE, it turned out that they predicted about a third of the variance in FLCA (a large effect size) but only a tenth of variance in FLE (a medium effect size). This can be used to further the argument that FLE and FLCA might be weakly correlated, but that they are definitely separate emotions (c.f., Dewaele and MacIntyre, 2014, 2016). Moreover, an analysis of participants’ stories about episodes of FLE and FLCA in class confirmed the statistical findings: they attributed FLE most often to the teacher while FLCA was mostly frequently linked to themselves.

In the latest development to improve the psychometric properties of the FLE questionnaire, Botes et al. (2019, unpublished) used exploratory and confirmatory factor analysis to re-analyze the dataset from Dewaele and MacIntyre (2014). A five-factor solution emerged, explaining close to half of the variance, with a first factor (FLE) that the majority of the items loaded onto and which explained close to a third of the variance. The items loading onto the next four factors were Personal Enjoyment, Social Enjoyment, Scholarly Enjoyment, and Teacher Appreciation. Botes et al. (2019, unpublished) concluded that FLE consists of a higher order general FLE factor with four first-order factors.

Budzińska (2018) pointed out that PP-inspired research in applied linguistics has privileged the first two pillars of PP, namely positive emotions and positive character traits, while the third pillar, the role of positive institutions, has been under-researched. Her ethnographic study of a Polish language school showed that the presence of happy and highly dedicated teachers who invested in their students’ linguistic progress, as well as their emotional well-being, created an upward spiral for the institution. The institution did not seek to reduce negativity, but focused on expanding positivity (p. 51).

#### Teacher-Focused Studies

PP-influenced research also emerged in the field of FL teacher emotions with edited books and special issues (Agudo, 2018; De Costa et al., 2018; Mercer and Kostoulas, 2018; Gkonou et al., in press). Researchers focused on the many threats to teachers’ emotional well-being (including teacher-internal variables such as their personality, classroom-specific variables such as the behavior of student, and wide contextual variables such as the institution or the national education system).

Mercer et al. (2016) and Talbot and Mercer (2018) focused on language teachers’ emotional well-being and the regulation strategies they use to manage their emotions in challenging situations. Similarly, Morris and King (2018) looked at how seven Japanese university teachers use emotion regulation strategies to deal with student apathy, classroom silence, misbehavior and difficult working conditions. In a follow-up study on the same database, Morris and King (in press) combined semi-structured interviews, classroom observations, and stimulated recall sessions to understand the dynamic interplay between context and their teachers’ regulation of their own and their students’ emotions, especially the negative ones, in order to fulfill their responsibilities, their teaching, and the maintenance of their own psychological well-being.

Emotion regulation was also at the heart of Oxford’s investigation (in press) into five teachers and teacher educators’ dynamic use of empathy, emotional intelligence, emotion regulation, and emotional labor to develop a “compass of emotion,” comprising both positive and negative emotions, allowing them to strengthen their emotional well-being.

Trait Emotional Intelligence (TEI) was also a central independent variable in Dewaele and Mercer’s (2018) study on 513 EFL/ESL teachers. Participants with higher levels of TEI reported significantly more positive attitudes toward students and enjoyed lively students more. Further research on the same database showed that higher levels of TEI were linked with better self-reported classroom management, pedagogical skills, and creativity (Dewaele and Li, 2018; Dewaele, 2018; Dewaele et al., 2018a). Dewaele (in press) found that teachers with high TEI were significantly more intrinsically motivated, had stronger identified regulation, and were less amotivated.

Li and Rawal (2018) showed that love toward the profession sustained a mathematics teacher in China and an English teacher in Nepal. Mutual love, understanding, and support between teachers and students was vital during classroom interactions and helped the teachers avoid being dragged down by work-related sociopolitical factors. A case study of a veteran English lecturer in China showed that Buddhist faith had a “transformative influence on both her emotional experiences and her identity development” (Ding and De Costa, 2018).

MacIntyre et al. (2019b) focused on the statistical relationships between 47 EFL teachers’ levels of well-being, perceptions of stress, and personality profiles. They used the PERMA profiler questionnaire and a big five-personality questionnaire, the International Personality Item Pool (IPIP). They found that Emotional Stability was most strongly correlated with the PERMA dimensions. The correlation was positive and particularly strong with positive emotions (35% of shared variance) and was negatively correlated with negative emotions (31% of shared variance). The PERMA well-being score correlated significantly with four of the five personality traits (Agreeableness, Conscientiousness, Emotional Stability and Intellect). Only Extraversion did not correlate with teacher well-being. Considering the most frequent chronic stressors in their professional lives, participants listed heavy workload, financial stress, and long hours. Financial difficulties also topped the list of life event stressors in the past year.

#### Intervention Studies

The interventions in the current period are focused less on linguistic outcomes and more on learners’ (and teachers’) well-being, engagement, agentic feelings, emotional awareness, sense of control over their lives and ability to surmount obstacles. Such a mindset can play a crucial role in linguistic development for learners and professional development for teachers (Fresacher, 2016; Gabryś-Barker, 2016; Guz and Tetiurka, 2016; Helgesen, 2016; Hiver, 2016; Mercer, 2016; Oxford, 2016b). Interventions can include poetry (Piasecka, 2016); music (Fonseca-Mora and Herrero Machancoses, 2016), walking in the classroom space music (Mitchell et al., 2019), a combination of music, laughter, gratitude (Gregersen et al., 2016), gratitude, altruism, music, pets, exercise and laughter (Gregersen, 2016) or even helping others in order in order to get out of a self-focus (Murphey, 2016). Gregersen et al. (in press) used a cognitive reappraisal strategy during a moderately successful weeklong intervention with a single teacher-participant attempting to limit stress by highlighting the “silver linings.”

In the concluding chapter of the book *Emotional Rollercoaster of Language Teaching*, King et al. (in press) point out that teachers’ emotional resilience can be strengthened firstly by raising awareness of their own emotions and that of their students and, secondly, by helping them develop strategies to avoid stress and diminish the longer-term risk of burnout. Teachers, like martial artists, need a strong control of body and mind, and sufficient physical and mental resilience to face challenges in the classroom and in the institution (c.f., Benesch, 2017), in order to defend themselves if needed, and to remain humble and realistic about their skills and achievements. King et al. (in press) conclude that emotional regulation needs to be included in teacher education, both pre-service and in-service.

#### Critical Considerations

Most of the studies that incorporated constructs from PP in this second period aimed at understanding variation at an individual or at a group level in order to optimize students’ or teachers’ strengths and well-being, and to improve foreign language teaching and learning, and assessment as a result. Overall, it seems that researchers paid heed to MacIntyre and Mercer’s (2014) exhortations, as studies in this period focused on emotions over different time-scales, confirmed the existence of ambivalent emotions, took into account the importance of contexts ranging from classroom to institution to country, and adopted a wide range of epistemological stances and methodological approaches. The influence of dynamic system theory permeated the field, with plenty of evidence emerging that learner and teacher emotions fluctuate dynamically depending on interactions between internal and external factors and may change over the longer term. Also, the number of studies adopting individual-level perspectives seems to be larger than those with group-level perspectives in the field of teacher emotions.

Some issues that have been pointed out in the past remain relevant today, including the danger of oversimplification of complex interacting processes in FL learning, such as telling unprepared FL learners that positive feelings are “the only path to greater proficiency” (Komorowska, 2016, p. 39). This danger is linked to the fact that research designs can only have a finite number of dependent and independent variables (c.f., Dörnyei, 2007) that can obscure the full panorama of interacting variables lurking in the background. Considering the second period, there seems to be a general trend toward more robustness, with development of psychometrically sound instruments and use of triangulation to zoom in on interesting phenomena. With cross-sectional studies dominating the field, it is clear that more longitudinal designs are needed in order to draw clearer causational conclusions (c.f., Lazarus, 2003). Also, case studies provide great insights but offer little generalizability (c.f., Dörnyei, 2007). We also feel that more PP-inspired interventional studies are needed in FL classrooms, using a wide variety of approaches, that seek ways to boost learners’ linguistic skills as well as their well-being (MacIntyre et al., 2019a).

## Conclusion

Providing a comprehensive overview of a field in full expansion is as difficult as trying to document the flowering of a thousand flowers, bushes, and fruit trees in a country park in spring sunshine, armed with a single camera. Inevitably, the views will be influenced by the photographer’s knowledge of the lay out of the park and the actual journey through it. It will be further shaped by the photographer’s predilection for certain spots, for certain colors, for certain contrasts between light and shade, for the presence or absence of water, of mist, of wildlife, or other visitors in the pictures. It is safe to assume that no two photographers would return home with identical pictures from the same park on the same day. While some central features would undoubtedly figure in the resulting albums of both photographers, composition, lighting, and perspective of pictures would be different. Moreover, because of restrictions on the maximum number of pictures, though decisions would have to be made on what to include and what to leave out. An album with 1,000 thumbnail pictures might be less attractive than one with fewer, but larger pictures. The commercial photo album promoting such a country park would not claim to be exhaustive, but would aim to raise awareness of its existence and maybe attract visitors to the park. Similarly, the current overview and special issue that it is part of aim to raise the profile of this emerging field of PP in applied linguistics in order to encourage teachers, students, and researchers to take a look and maybe to join us in the joyful quest for a better understanding of the complex workings of learners’ and teachers’ mind and hearts.

## Author Contributions

J-MD, XC, AP, and JL contributed to research design and literature review.

### Conflict of Interest Statement

The authors declare that the research was conducted in the absence of any commercial or financial relationships that could be construed as a potential conflict of interest.

## Footnotes

^1^A description of the chronological development of PP lies beyond the scope of the present paper.

^2^The two papers are identical.

## References

[ref300] Agudo J. D. D. M. (ed.) (2018). “Emotions in second language teaching” in Theory, research and teacher education. Switzerland: Springer.

[ref1] Aguilar-Río J. I. (2013). L’enseignement d’une langue comme pratique émotionnelle: caractérisation d’une performance, ébauche d’une competence. Lidil 48, 137–156. 10.4000/lidil.3323

[ref2] Arnold J. (ed.) (1999). Affect in language learning. Cambridge: Cambridge University Press.

[ref3] Arnold J. Fonseca C. (2007). “Affect in teacher talk” in Language acquisition and development. ed. Tomlinson B. (London: Continuum), 107–121.

[ref4] Arnold J. Fonseca Mora C. (2011). Introduction: an affective perspective on language learning and teaching. Anglistik: Int. J. Engl. Stud. 22, 7–9.

[ref5] Avila-López J. (ed.) (2015). “Didáctica de la emoción: De la investigación al aula de ELE” in MARCO ELE: Revista de Didáctica Español Lengua Extranjera, 21.

[ref7] Belnap R. C. Bown J. Dewey D. P. Belnap L. P. Steffen P. R. (2016). “Project perseverance: helping students become self-regulating learners” in Positive psychology in SLA. eds. MacIntyre P. D. Gregersen T. Mercer S. (Bristol: Multilingual Matters), 282–304.

[ref8] Benesch S. (2017). Emotions and English language teaching: Exploring teachers’ emotion labor. NY: Routledge.

[ref9] Benesch S. (2018). Commentary. In H. Rawal, P. De Costa & Wendy Li (eds.), emotions in second language teaching: theory, research and teacher education. [Special issue]. Chin. J. Appl. Linguist. 41, 571–577. 10.1515/cjal-2018-0039

[ref10] Berdal-Masuy F. Pairon J. (2019). Emo-langage: Vers une approche transversale des langages dans leurs dynamiques émotionnelles et creatives (Emo-language: Towards a transversal approach to languages in their emotional and creative dynamics). [Special Issue]. TIPA 35.

[ref11] Boudreau C. MacIntyre P. D. Dewaele J.-M. (2018). Enjoyment and anxiety in second language communication: an idiodynamic approach. Stud. Second Lang. Learn. Teach. 8, 149–170. 10.14746/ssllt.2018.8.1.7

[ref12] Bown J. White C. J. (2010). Affect in a self-regulatory framework for language learning. System 38, 432–443. 10.1016/j.system.2010.03.016

[ref13] Budzińska K. (2018). Positive institutions: a case study. Theory Pract. Second Lang. Acquis. 4, 33–54.

[ref14] Chaffee K. E. Noels K. A. McEown M. S. (2014). Learning from authoritarian teachers: controlling the situation or controlling yourself can sustain motivation. Stud. Second Lang. Learn. Teach. 4, 355–387. 10.14746/ssllt.2014.4.2.9

[ref15] Chen X. Padilla A. M. (2019). New orientation in bilingual research: A study of flourishing using a positive psychology perspective. Atlanta, U.S.A: The American Applied Linguistics Association (AAAL) 9–12 March, 2019.

[ref16] Chen X. Padilla A. M. Vallerand J. R. (2019). The facilitative role of two types of passion in second language learning. In: The 126th Annual Conference of American Psychological Association (APA). Chicago, USA 8–11 August, 2019

[ref17] Csikszentmihalyi M. Nakamura J. (2011). “Positive psychology: where did it come from, where is it going?” in Designing positive psychology: Taking stock and moving forward. eds. Sheldon M. K. Kashdan T. B. Steger M. F. (New York: Oxford University Press), 3–8.

[ref18] Czimmermann E. Piniel K. (2016). “Advanced language learners’ experiences of flow in the Hungarian EFL classroom” in Positive psychology in SLA. eds. MacIntyre P. D. Gregersen T. Mercer S. (Bristol: Multilingual Matters), 193–214.

[ref19] De Costa P. I. Rawal H. Li W. (2018). Broadening the second language teacher education agenda: international perspectives on teacher emotions. Chin. J. Appl. Linguist. 41, 401–409. 10.1515/cjal-2018-0030

[ref20] De Smet A. Mettewie L. Galand B. Hiligsmann P. Van Mensel L. (2018). Classroom anxiety and enjoyment in CLIL and non-CLIL: does the target language matter? Stud. Second Lang. Learn. Teach. 8, 47–72. 10.14746/ssllt.2018.8.1.3

[ref21] Dewaele J.-M. (2005). Investigating the psychological and the emotional dimensions in instructed language learning: obstacles and possibilities. Mod. Lang. J. 89, 367–380. 10.1111/j.1540-4781.2005.00311.x

[ref22] Dewaele J.-M. (2015). On emotions in foreign language learning and use. Lang. Teach. 39, 13–15.

[ref23] Dewaele J. M. Li C. (2018). Emotions in SLA [Special issue]. Stud. Second Lang. Learn. Teach. 8, 15–19. 10.14746/ssllt.2018.8.1.1

[ref24] Dewaele J.-M. (2018). The relationship between trait emotional intelligence and experienced ESL/EFL teachers’ love of English, attitudes towards their students and institution, self-reported classroom practices, enjoyment and creativity. In H. Rawal, P. De Costa, & W. Li (eds.), emotions in second language teaching: theory, research and teacher education. [Special issue]. Chin. J. Appl. Linguist. 41, 468–487. 10.1515/cjal-2018-0023

[ref25] Dewaele J.-M. (2019a). When elephants fly: the lift-off of emotion research in applied linguistics. In M. Bigelow (ed.), perspectives: (re)considering the role of emotion in language teaching and learning. [Special issue]. Mod. Lang. J. 103, 533–536. 10.1111/modl.12576

[ref26] Dewaele J.-M. (2019b). The effect of classroom emotions, attitudes toward English, and teacher behaviour on willingness to communicate among English foreign language learners. J. Lang. Soc. Psychol. 38, 523–535. 10.1177/0261927X19864996

[ref27] Dewaele J.-M. (in press). “What psychological, linguistic and sociobiographical variables power EFL/ESL teachers’ motivation?” in Language teaching: An emotional rollercoaster. eds. Gkonou C. Dewaele J.-M. King J. (Bristol: Multilingual Matters).

[ref28] Dewaele J.-M. Alfawzan M. (2018). Does the effect of enjoyment outweigh that of anxiety in foreign language performance? Stud. Second Lang. Learn. Teach. 8, 21–45. 10.14746/ssllt.2018.8.1.2

[ref29] Dewaele J.-M. Dewaele L. (2017). The dynamic interactions in foreign language classroom anxiety and foreign language enjoyment of pupils aged 12 to 18. A pseudo-longitudinal investigation. J. Eur. Second Lang. Assoc. 1, 12–22. 10.22599/jesla.6

[ref30] Dewaele J.-M. Dewaele L. (2018). Learner-internal and learner-external predictors of willingness to communicate in the FL classroom. J. Eur. Second Lang. Assoc. 2, 24–37. 10.22599/jesla.37

[ref31] Dewaele J.-M. Dewaele L. (in press). Is foreign language enjoyment more fleeting than foreign language classroom anxiety? An investigation into the dynamics of learners’ classroom emotions. Stud. Second Lang. Learn. Teach.

[ref32] Dewaele J.-M. Franco Magdalena A. Saito K. (2019a). The effect of perception of teacher characteristics on Spanish EFL learners’ anxiety and enjoyment. Mod. Lang. J. 103, 412–427. 10.1111/modl.12555

[ref33] Dewaele J.-M. Gkonou C. Mercer S. (2018a). “Do ESL/EFL teachers’ emotional intelligence, teaching experience, proficiency and gender, affect their classroom practice?” in Emotions in second language teaching. ed. de Dios Martínez Agudo J. (Berlin: Springer), 125–141.

[ref34] Dewaele J.-M. MacIntyre P. D. (2014). The two faces of Janus? Anxiety and enjoyment in the foreign language classroom. Stud. Second Lang. Learn. Teach. 4, 237–274. 10.14746/ssllt.2014.4.2.5

[ref35] Dewaele J.-M. MacIntyre P. D. (2016). “Foreign language enjoyment and foreign language classroom anxiety. The right and left feet of FL learning?” in Positive psychology in SLA. eds. MacIntyre P. D. Gregersen T. Mercer S. (Bristol: Multilingual Matters), 215–236.

[ref36] Dewaele J.-M. MacIntyre P. D. (2019). “The predictive power of multicultural personality traits, learner and teacher variables on foreign language enjoyment and anxiety” in Evidence-based second language pedagogy: A collection of instructed second language acquisition studies. eds. Sato M. Loewen S. (London: Routledge), 263–286.

[ref37] Dewaele J.-M. MacIntyre P. D. (in press). “El flujo en el aula de español como lengua extranjera (Flow in the Spanish foreign language classroom)” in Factores cognitivos y afectivos en la enseñanza de español como lengua extranjera. eds. del Carmen Méndez M. Andoni Duñabeitia J. (London: Routledge).

[ref38] Dewaele J.-M. Mercer S. (2018). “Variation in ESL/EFL teachers’ attitudes towards their students” in Teacher psychology in SLA. eds. Mercer S. Kostoulas A. (Bristol: Multilingual Matters), 178–195.

[ref39] Dewaele J.-M. Özdemir C. Karci D. Uysal S. Özdemir E. D. Balta N. (2019b). How distinctive is the foreign language enjoyment and foreign language classroom anxiety of Kazakh learners of Turkish? Appl. Linguist. Rev. 10.1515/applirev-2019-0021

[ref40] Dewaele J.-M. Witney J. Saito K. Dewaele L. (2018b). Foreign language enjoyment and anxiety in the FL classroom: the effect of teacher and learner variables. Lang. Teach. Res. 22, 676–697. 10.1177/1362168817692161

[ref41] Dewey D. Belnap R. K. Steffen P. (2018). Anxiety: stress, foreign language classroom anxiety, and enjoyment during study abroad in Amman, Jordan. Annu. Rev. Appl. Linguist. 38, 140–161. 10.1017/S0267190518000107

[ref42] Diener E. (2009). “Positive psychology: past, present and future” in Oxford handbook of positive psychology. eds. Snyder C. R. Lopez S. J. (New York, NY, US: Oxford University Press), 7–11.

[ref43] Ding X. De Costa P. I. (2018). Faith–based teacher emotional experiences: a case study of a veteran English lecturer in China. Chin. J. Appl. Linguist. 41, 532–551. 10.1515/cjal-2018-0037

[ref44] Dörnyei Z. (2007). Research methods in applied linguistics: Quantitative, qualitative and mixed methodologies. Oxford: Oxford University Press.

[ref45] Egbert J. (2003). A study of flow theory in the foreign language classroom. Mod. Lang. J. 87, 499–517. 10.1111/1540-4781.00204

[ref46] Egbert J. (2004). A study of flow theory in the foreign language classroom. Can. Mod. Lang. Rev. 60, 549–586. 10.3138/cmlr.60.5.549

[ref47] Falout J. (2014). Circular seating arrangements: approaching the social crux in language classrooms. Stud. Second Lang. Learn. Teach. 4, 275–300. 10.14746/ssllt.2014.4.2.6

[ref48] Falout J. (2016). “The dynamics of past selves in language learning and well-being” in Positive psychology in SLA. eds. MacIntyre P. D. Gregersen T. Mercer S. (Bristol: Multilingual Matters), 112–129.

[ref49] Fonseca-Mora M. C. Herrero Machancoses F. (2016). “Music and language learning: emotions and engaging memory pathways” in Positive psychology in SLA. eds. MacIntyre P. D. Gregersen T. Mercer S. (Bristol: Multilingual Matters), 359–373.

[ref50] Fredrickson B. L. (2001). The role of positive emotions in positive psychology: the broaden-and-build theory of positive emotions. Am. Psychol. 56, 218–226. 10.1037/0003-066X.56.3.218, PMID: 11315248 PMC3122271

[ref51] Fredrickson B. L. (2003). The value of positive emotions. Am. Sci. 91, 330–335. 10.1511/2003.4.330

[ref52] Fredrickson B. L. (2006). “The broaden-and-build theory of positive emotions” in A life worth living: Contributions to positive psychology. eds. Csikszentmihalyi M. Csikszentmihalyi I. S. (New York: Oxford University Press), 85–103.

[ref53] Fresacher C. (2016). “Why and how to use positive psychology activities in the second language classroom” in Positive psychology in SLA. eds. MacIntyre P. D. Gregersen T. Mercer S. (Bristol: Multilingual Matters), 344–358.

[ref54] Frijda N. H. (1986). The emotions. Cambridge: Cambridge University Press.

[ref55] Gabryś-Barker D. (2014). Success: from failure to failure with enthusiasm. Stud. Second Lang. Learn. Teach. 4, 301–325. 10.14746/ssllt.2014.4.2.7

[ref56] Gabryś-Barker D. (2016). “Caring and sharing in the foreign language class: on a positive classroom climate” in Positive psychology perspectives on foreign language learning and teaching. eds. Gabryś-Barker D. Gałajda D. (New York: Springer), 155–174.

[ref57] Gabryś-Barker D. Gałajda D. (eds.) (2016). Positive psychology perspective on foreign language learning and teaching. New York: Springer.

[ref58] Gkonou C. Dewaele J.-M. King J. (eds.) (in press). The emotional rollercoaster of language teaching. Bristol: Multilingual Matters.

[ref59] Gregersen T. (2013). “Language learning vibes: what, why and how to capitalize for positive affect” in The affective dimension in second language acquisition. eds. Gabryś-Barker D. Bielska J. (Bristol, UK: Multilingual Matters), 89–98.

[ref60] Gregersen T. (2016). “The positive broadening power of a focus on well-being in the language classroom” in Positive psychology perspectives on foreign language learning and teaching. eds. Gabryś-Barker D. Gałajda D. (New York: Springer), 59–73.

[ref61] Gregersen T. MacIntyre P. D. Finegan K. H. Talbot K. R. Claman S. L. (2014). Examining emotional intelligence within the context of positive psychology interventions. Stud. Second Lang. Learn. Teach. 4, 327–353. 10.14746/ssllt.2014.4.2.8

[ref63] Gregersen T. MacIntyre P. Meza M. (2016). “Positive psychology exercises build social capital for language learners: preliminary evidence” in Positive psychology in SLA. eds. MacIntyre P. D. Gregersen T. Mercer S. (Bristol: Multilingual Matters), 147–167.

[ref64] Gregersen T. MacIntyre P. Macmillan N. (in press). “Dealing with the emotions of teaching abroad: searching for silver linings in a difficult context” in The emotional rollercoaster of language teaching. eds. Gkonou C. Dewaele J.-M. King J. (Bristol: Multilingual Matters)

[ref65] Guz E. Tetiurka M. (2016). “Positive emotions and learner engagement: insights from an early FL classroom” in Positive psychology perspectives on foreign language learning and teaching. eds. Gabryś-Barker D. Gałajda D. (New York: Springer), 133–153.

[ref66] Helgesen M. (2016). “Happiness in ESL/EFL: bringing positive psychology to the classroom” in Positive psychology in SLA. eds. MacIntyre P. D. Gregersen T. Mercer S. (Bristol: Multilingual Matters), 305–323.

[ref67] Hiver P. (2016). “The triumph over experience: Hope and hardiness in novice L2 teachers” in Positive psychology in SLA. eds. MacIntyre P. D. Gregersen T. Mercer S. (Bristol: Multilingual Matters), 168–192.

[ref69] Horwitz E. Horwitz M. Cope J. (1986). Foreign language classroom anxiety. Mod. Lang. J. 70, 125–132. 10.1111/j.1540-4781.1986.tb05256.x

[ref70] Ibrahim Z. (2016). “Affect in directed motivational currents: positive emotionality in long-term L2 engagement” in Positive psychology in SLA. eds. MacIntyre P. D. Gregersen T. Mercer S. (Bristol: Multilingual Matters), 258–281.

[ref71] Jiang Y. Dewaele J. M. (2019). How unique is the foreign language classroom enjoyment and anxiety of Chinese EFL learners? System 82, 13–25. 10.1016/j.system.2019.02.017

[ref72] Jin Y. X. Dewaele J. M. (2018). The effect of positive orientation and perceived social support on foreign language classroom anxiety. System 74, 149–157. 10.1016/j.system.2018.01.002

[ref73] Jin Y. X. Jun Zhang L. (2018). The dimensions of foreign language classroom enjoyment and their effect on foreign language achievement. Int. J. Biling. Educ. Biling. 10.1080/13670050.2018.1526253 Online first

[ref74] Jin Y. X. Jun Zhang L. (2019). A comparative study of two scales for foreign language classroom enjoyment. Percept. Mot. Skills. 10.1177/0031512519864471, PMID: 31345113

[ref75] King J. Dewaele J.-M. Gkonou C. (in press). “Concluding thoughts on the emotional rollercoaster of language teaching” in The emotional rollercoaster of language teaching. eds. Gkonou C. Dewaele J.-M. King J. (Bristol: Multilingual Matters).

[ref76] Komorowska H. (2016). “Difficulty and coping strategies in language education: is positive psychology misrepresented in SLA/FLT?” in Positive psychology perspectives on foreign language learning and teaching. eds. Gabryś-Barker D. Gałajda D. (Cham: Springer), 39–56.

[ref77] Kusiak-Pisowacka M. (2016). “How to test for the best: implementing positive psychology in foreign language testing” in Positive psychology perspectives on foreign language learning and teaching. eds. Gabryś-Barker D. Gałajda D. (Cham: Springer), 289–306.

[ref78] Lake J. (2013). “Positive L2 self: linking positive psychology with L2 motivation” in Language learning motivation in Japan. eds. Apple M. Da Silva D. Fellner T. (Bristol: Multilingual Matters), 225–244.

[ref79] Lake J. (2016). “Accentuate the positive: conceptual and empirical development of the positive L2 self and its relationship to L2 proficiency” in Positive psychology in SLA. eds. MacIntyre P. D. Gregersen T. Mercer S. (Bristol: Multilingual Matters), 237–257.

[ref80] Lantolf J. Swain M. (2019). On the emotion ~ cognition dialectic: a sociocultural response to prior. In M. Bigelow (ed.), perspectives: (re)considering the role of emotion in language teaching and learning. [Special issue]. Mod. Lang. J. 103, 528–530. 10.1111/modl.12574

[ref81] Lazarus R. S. (2003). Does the positive psychology movement have legs? Psychol. Inq. 14, 93–109. 10.1207/S15327965PLI1402_02

[ref82] Li C. (ed.). (in press). A positive psychology perspective on emotions in SLA [Special issue]. Foreign Lang. World.

[ref83] Li C. Dewaele J.-M. Jiang G. (2019). The complex relationship between classroom emotions and EFL achievement in China. Appl. Linguist. Rev. 10.1515/applirev-2018-0043 Online first

[ref84] Li C. Jiang G. Dewaele J.-M. (2018). Understanding Chinese high school students’ foreign language enjoyment: Validation of the Chinese version of the Foreign Language Enjoyment Scale. System 76, 183–196. 10.1016/j.system.2018.06.004

[ref85] Li W. Rawal H. (2018). Waning and waxing of love: unpacking layers of teacher emotion. Chin. J. Appl. Linguist. 41, 552–570. 10.1515/cjal-2018-0038

[ref86] Lopez S. J. Gallagher M. W. (2009). “A case for positive psychology” in Oxford handbook of positive psychology. eds. Lopez S. J. Snyder C. R. (Oxford: Oxford University Press).

[ref87] Lopez S. J. Snyder C. R. (2009). Oxford handbook of positive psychology. Oxford: Oxford University Press.

[ref90] MacIntyre P. D. Gregersen T. (2012). Emotions that facilitate language learning: the positive-broadening power of the imagination. Stud. Second Lang. Learn. Teach. 2, 193–213. 10.14746/ssllt.2012.2.2.4

[ref91] MacIntyre P. D. Gregersen T. Mercer S. (2016). “Conclusion” in Positive psychology in SLA. eds. MacIntyre P. D. Gregersen T. Mercer S. (Bristol: Multilingual Matters), 374–379.

[ref92] MacIntyre P. D. Gregersen T. Mercer S. (2019a). Setting an agenda for positive psychology in SLA: theory, practice, and research. Mod. Lang. J. 103, 262–274. 10.1111/modl.12544

[ref93] MacIntyre P. D. Mackinnon S. P. Clément R. (2009). “Embracing affective ambivalence: a research agenda for understanding the interdependent processes of language anxiety and motivation” in Cultural identity and language anxiety. eds. Cheng P. Yan J. X. (Guilin, China: Guangxi Normal University Press), 3–34.

[ref94] MacIntyre P. D. Mercer S. (2014). Introducing positive psychology to SLA. Stud. Second Lang. Learn. Teach. 4, 153–172. 10.14746/ssllt.2014.4.2.2

[ref96] MacIntyre P. D. Ross J. Talbot K. Gregersen T. Mercer S. Banga C. A. (2019b). Stressors, personality and wellbeing among language teachers. System 82, 26–38. 10.1016/j.system.2019.02.013

[ref97] Mackenzie, J. L. Alba Juez, L. (eds.) (2019). Emotion in discourse. Amsterdam: Benjamins.

[ref98] Mercer S. (2016). “Seeing the world through your eyes: empathy in language learning and teaching” in Positive psychology in SLA. eds. MacIntyre P. D. Gregersen T. Mercer S. (Bristol: Multilingual Matters), 91–111.

[ref99] Mercer, S. Kostoulas, A. (eds.) (2018). Teacher psychology in SLA. Bristol: Multilingual Matters.

[ref100] Mercer S. Oberdorfer P. Saleem M. (2016). “Helping language teachers to thrive: using positive psychology to promote teachers’ professional well-being” in Positive psychology perspectives on foreign language learning and teaching. eds. Gabryś-Barker D. Gałajda D. (Cham: Springer), 213–229.

[ref101] Mitchell C. Guy R. Fournel A. Treille A. De Koning M. (2019). Apprentissage affectif pour une communication effective: l’émotion et la prosodie dans une démarche d’improvisation théâtrale (affective learning for effective communication: emotion and prosody in theatre improvisation). TIPA 35, 1–19. 10.4000/tipa.3297

[ref102] Morris S. King J. (2018). Teacher frustration and emotion regulation in university language teaching. Chin. J. Appl. Linguist. 41, 433–452. 10.1515/cjal-2018-0032

[ref103] Morris S. King J. (in press). “Emotion regulation amongst university EFL teachers in Japan: the dynamic interplay between context and emotional behaviour” in The emotional rollercoaster of language teaching. eds. Gkonou C. Dewaele J.-M. King J. (Bristol: Multilingual Matters)

[ref104] Murphey T. (2014). Singing well-becoming: student musical therapy case studies. Stud. Second Lang. Learn. Teach. 4, 205–235. 10.14746/ssllt.2014.4.2.4

[ref105] Murphey T. (2016). “Teaching to learn and well-become: many mini-renaissances” in Positive psychology in SLA. eds. MacIntyre P. D. Gregersen T. Mercer S. (Bristol: Multilingual Matters), 324–343.

[ref107] Oxford R. (2016a). “Toward a psychology of well-being for language learners: the ‘EMPATHICS’ vision” in Positive psychology in SLA. eds. MacIntyre P. D. Gregersen T. Mercer S. (Bristol: Multilingual Matters), 10–90.

[ref108] Oxford R. L. (2016b). “Powerfully positive: searching for a model of language learner well-being” in Positive psychology perspectives on foreign language learning and teaching. eds. Gabryś-Barker D. Gałajda D. (New York: Springer), 21–37.

[ref109] Oxford R. L. (2017). “Anxious language learners can change their minds: ideas and strategies from traditional psychology and positive psychology” in New insights into language anxiety: Theory, research and educational implications. eds. Gkonou C. Daubney M. Dewaele J.-M. (Bristol: Multilingual Matters), 179–199.

[ref110] Oxford R. L. (in press). “The well of language teachers’ emotional well-being” in The emotional rollercoaster of language teaching. eds. Gkonou C. Dewaele J.-M. King J. (Bristol: Multilingual Matters)

[ref111] Oxford R. Cuéllar L. (2014). Positive psychology in cross-cultural narratives: Mexican students discover themselves while learning Chinese. Stud. Second Lang. Learn. Teach. 4, 173–203. 10.14746/ssllt.2014.4.2.3

[ref112] Pavelescu L. Petrić B. (2018). Love and enjoyment in context: four case studies of adolescent EFL learners. Stud. Second Lang. Learn. Teach. 8, 73–101. 10.14746/ssllt.2018.8.1.4

[ref113] Pavlenko A. (2013). “The affective turn in SLA: from ‘affective factors’ to ‘language desire’ and ‘commodification of affect’” in The affective dimension in second language acquisition. eds. Gabryś-Barker D. Bielska J. (Bristol, UK: Multilingual Matters), 3–28.

[ref114] Peterson C. (2006). A primer in positive psychology. New York: Oxford University Press.

[ref115] Piasecka L. (2016). “Activating character strengths though poetic encounters in a foreign language—a case study” in Positive psychology perspectives on foreign language learning and teaching. eds. Gabryś-Barker D. Gałajda D. (New York: Springer), 75–92.

[ref116] Piechurska-Kuciel E. (2017). “L2 or L3? Foreign language enjoyment and proficiency” in Multiculturalism, multilingualism and the self. eds. Gabryś-Barker D. Gałajda D. Wojtaszek A. Zakrajewski P. (Cham: Springer), 97–111.

[ref118] Prior M. T. (2019). Elephants in the room: an “affective turn,” or just feeling our way? In M. Bigelow (ed.), perspectives: (re)considering the role of emotion in language teaching and learning. [Special issue]. Mod. Lang. J. 103, 516–527. 10.1111/modl.12573

[ref119] Puozzo Capron I. Piccardo E. (eds.) (2013). L’émotion et l’apprentissage des langues [Special Issue]. Lidil, 48, 5–16.

[ref120] Ross A. S. Rivers D. J. (2018). Emotional experiences beyond the classroom: interactions with the social world. Stud. Second Lang. Learn. Teach. 8, 103–126. 10.14746/ssllt.2018.8.1.5

[ref121] Rubio F. D. (2011). Optimal experiences in the foreign language classroom: flow states in speaking tasks. Anglistik: Int. J. Engl. Stud. 22, 63–79.

[ref122] Saito K. Dewaele J.-M. Abe M. In’nami Y. (2018). Motivation, emotion, learning experience and second language comprehensibility development in classroom settings. Lang. Learn. 68, 709–743. 10.1111/lang.12297

[ref123] Seligman M. (2018). PERMA and the building blocks of well-being. J. Posit. Psychol. 13, 333–335. 10.1080/17439760.2018.1437466

[ref124] Seligman M. E. P. Csikszentmihalyi M. (2000). Positive psychology: an introduction. Am. Psychol. 55, 5–14. 10.1037/0003-066X.55.1.5, PMID: 11392865

[ref125] Seligman M. Ernst R. M. Gillham J. Reivich K. Linkins M. (2009). Positive education: positive psychology and classroom interventions. Oxf. Rev. Educ. 35, 293–311. 10.1080/03054980902934563

[ref126] Sharwood Smith M. (2017). Introducing language and cognition. A map of the mind. Cambridge: Cambridge University Press.

[ref127] Swain M. (2013). The inseparability of cognition and emotion in second language learning. Lang. Teach. 46, 195–207. 10.1017/S0261444811000486

[ref128] Talbot K. Mercer S. (2018). Exploring university ESL/EFL teachers’ emotional well-being and emotional regulation in the United States, Japan and Austria. Chin. J. Appl. Linguist. 41, 410–432. 10.1515/cjal-2018-0031

[ref129] Tardy C. M. Snyder B. (2004). ‘That’s why I do it’: flow and EFL teachers’ practices. ELT J. 58, 118–128. 10.1093/elt/58.2.118

[ref130] White C. J. (2018). “Emotional turn in applied linguistics and TESOL: significance, challenges and prospects” in Emotions in second language teaching: Theory, research and teacher education. ed. de Dios Martínez Agudo J. (Berlin: Springer), 19–34.

